# Second Primary Lung Adenocarcinoma After Intensity-Modulated Radiotherapy for Nasopharyngeal Carcinoma

**DOI:** 10.3389/fonc.2022.801090

**Published:** 2022-02-24

**Authors:** Fen Xue, Xiaoshuang Niu, Chaosu Hu, Xiayun He

**Affiliations:** ^1^Department of Radiation Oncology, Fudan University Shanghai Cancer Center, Shanghai, China; ^2^Department of Oncology, Shanghai Medical College, Fudan University, Shanghai, China; ^3^Shanghai Key Laboratory of Radiation Oncology, Fudan University Shanghai Cancer Center, Shanghai, China

**Keywords:** nasopharyngeal carcinoma, intensity-modulated radiotherapy, second primary lung adenocarcinoma, cumulative incidence risk, survival

## Abstract

**Objective:**

The improvement of the efficacy of intensity-modulated radiotherapy (IMRT) for nasopharyngeal cancer (NPC) has prolonged the survival of patients, and the incidence of the second tumor has gradually increased. Among them, second primary lung adenocarcinoma (SPLAC) attributes the highest incidence. This study aimed to determine the long-term risk of SPLAC in NPC patients after IMRT.

**Methods:**

From May 2005 to May 2018, a total of 1,102 non-metastatic NPC patients who received IMRT in our hospital were enrolled, and the incidence and efficacy of SPLAC were followed up in the long term.

**Results:**

Over a median follow-up period of 66 months, a total of 22 cases of SPLAC were observed, with an incidence of 2.0%. The 1-, 2-, 3-, 4-, and 5-year cumulative risks of SPLAC were 0.4%, 0.7%, 0.8%, 1.1%, and 1.7%, respectively. During follow-up, 90.9% (20/22) of the SPLAC detected was in early stage, and the recurrence rate of surgery alone was 5.3% (1/19).

**Conclusion:**

In NPC patients, the proportion of SPLAC after IMRT was similar to that of the normal population, and most of them were found in early stage during follow-up, with good surgical efficacy.

## Introduction

Compared to the era of 2-dimensional radiotherapy (2DRT), the efficacy of treatment for nasopharyngeal carcinoma (NPC) has been significantly improved by intensity-modulated radiotherapy (IMRT), and IMRT has now become the main treatment for NPC. It has been reported that the 10-year overall survival (OS) rate of NPC patients after IMRT is about 72.6%–75.0% (1, 2). The main reasons impairing long-term survival were distant metastasis and locoregional recurrence, with 10-year local failure-free survival (LFFS), regional failure-free survival (RFFS), and distant metastasis-free survival (DMFS) around 89.0%–90.0%, 95.0%–95.9%, and 79.8%–83.3% according to the literature. While the second tumor is also an important reason (1–5). The incidence of the second primary tumor after IMRT in NPC patients was 3.0%–9.2%, with second primary lung adenocarcinoma (SPLAC) contributing the highest incidence (6, 7). With the prolongation of survival, the incidence of the second primary tumor gradually increased. Zhang et al. (6) conducted a long-term follow-up study of 6,377 NPC patients who received IMRT and found that 189 (3.0%) patients developed the second primary tumor. The 1-, 2-, 3-, 4-, and 5-year cumulative risks of second primary tumor were 0.4%, 0.9%, 1.6%, 2.2% and 2.6%, respectively. Among them, lung cancer had the highest incidence (50/6,377, 0.78%), followed by oral cancer, liver cancer, colorectal cancer, and thyroid cancer. According to the results of a chest low-dose computed tomography (LDCT) screening study in China (8), the proportion of lung squamous cell carcinoma was relatively low and lung adenocarcinoma and disease with early stage (0/I) are relatively high, which suggested that more attention were needed for distinguishing SPLAC from lung metastasis of NPC. At present, there are rare reports about the incidence and outcome of SPLAC after IMRT for NPC. Therefore, we conducted this retrospective study to compare the difference of SPLAC incidence and outcome between NPC survivors after treatment and the general population.

## Patients and Methods

### Patient Selection and Evaluation

From May 2005 to May 2018, 1,102 patients with newly diagnosed, pathologically proven, non-metastatic, previously untreated NPC treated with IMRT ± chemotherapy at Fudan University Shanghai Cancer Center were retrospectively enrolled. The exclusion criteria were as follows: 1) pathologically proven non-squamous cell carcinoma; 2) history of previous malignancy before NPC diagnosis or other concomitant malignancy; 3) incomplete clinicopathologic and treatment data available; 4) incomplete radiotherapy. All patients were restaged according to the eighth edition of the International Union against Cancer/American Joint Committee on Cancer (UICC/AJCC) system. The diagnostic criteria of SPLAC were as follows: 1) histopathology- or cytology-proven SPLAC; 2) elimination of the possibility of metastasis from the primary tumor or other second primary tumor; 3) SPLAC occurrence at least after 6 months from IMRT completion.

Initial evaluation included a complete history and physical examination, blood routine and biochemistry tests, fiberoptic nasopharyngoscopy, pathological diagnosis of nasopharynx, enhanced magnetic resonance imaging (MRI) of the nasopharynx, enhanced MRI/CT of the neck. Other assessments included positron emission tomography-CT (PET-CT) or replaced by chest CT, abdominal ultrasound/CT, and bone emission CT. All patients underwent a multidisciplinary discussion before treatment.

### Treatment

All the patients received definitive IMRT. The primary gross tumor volume (GTV) included lesion of nasopharynx and positive lymph nodes. The prescribed doses were 66.0–70.4 Gy/30–32 fractions for the PTVp [the planning target volume (PTV) covering the GTV with an additional 5-mm margin]. Clinical target volume (CTV) included the PTVp, the nasopharynx, parapharyngeal space, posterior one-third of the nasal cavity and maxillary sinus, anterior part of clivus, pterygoid plate, pterygoid fossa, skull base, inferior sphenoid sinus, retropharyngeal lymph nodes, drainage region of the neck (levels II, III, and VA for N0 patients and levels II, III, IV, and VA-B for N1 patients). PTVc was created by expanding a 5-mm margin around the CTV to compensate for geometric uncertainties and patient movement. The prescribed doses were 60.0 and 54.0 Gy for high-risk PTVc and low-risk PTVc, respectively. All patients received five daily fractions per week.

Patients with stage I disease were not administered chemotherapy. Part of the patients with stage II disease and all patients with stage III–IVA disease received platinum-based chemotherapy, including concurrent chemoradiotherapy (CCRT) with or without neoadjuvant chemotherapy (IC)/adjuvant chemotherapy (AC). CCRT regimen included cisplatin 30–40 mg/m^2^/day on day 1 every week or cisplatin 80 mg/m^2^/day on day 1 every 3 weeks. IC and AC regimens included TPF regimen (docetaxel 60 mg/m^2^/day, day 1, cisplatin 25 mg/m^2^/day, days 1–3, and 5-fluorouracil 0.5 g/m^2^/day with a 120-h infusion, repeated every 3 weeks), PF regimen (cisplatin 25 mg/m^2^/day, days 1–3, and 5-fluorouracil 0.5 g/m^2^/day with a 120-h infusion, repeated every 3 weeks), and GP regimen (gemcitabine 1,000 mg/m^2^/day, days 1 and 8, and cisplatin 25 mg/m^2^/day, days 1–3, repeated every 3 weeks). Generally, IMRT was implemented 3 weeks after IC. AC was administered 4 weeks after the completion of radiotherapy for tolerable patients.

### Follow-Up and Evaluation

During the follow-up, patients were evaluated at 3-month intervals for the first 2 years, at 6-month intervals for the following 3 years, and then annually. Each follow-up visit included a complete history, physical examination, nasopharyngoscopy, an MRI scan of the nasopharynx, and MRI/CT scan of the neck. Chest CT and abdominal sonography/CT were conducted annually. Additional tests like bone scintigraphy were ordered whenever clinically indicated.

### Statistical Analysis

SPSS 26.0 (SPSS Inc, Chicago, IL, USA) was used for statistical analysis in this study. Statistical data were tested by χ^2^ test or by Fisher’s exact test if theoretical frequency T < 1 or n < 40. The actuarial LFFS, RFFS, DMFS, and OS were measured from the date of diagnosis to a documented event or the last follow-up visit. Cumulative incidence of SPLAC in the corresponding observed years and survival rates of patients were calculated using the Kaplan–Meier method and compared with log-rank test between different groups. A two-sided p-value <0.05 was statistically significant.

## Results

### Patient Demographics

**Figure 1:**
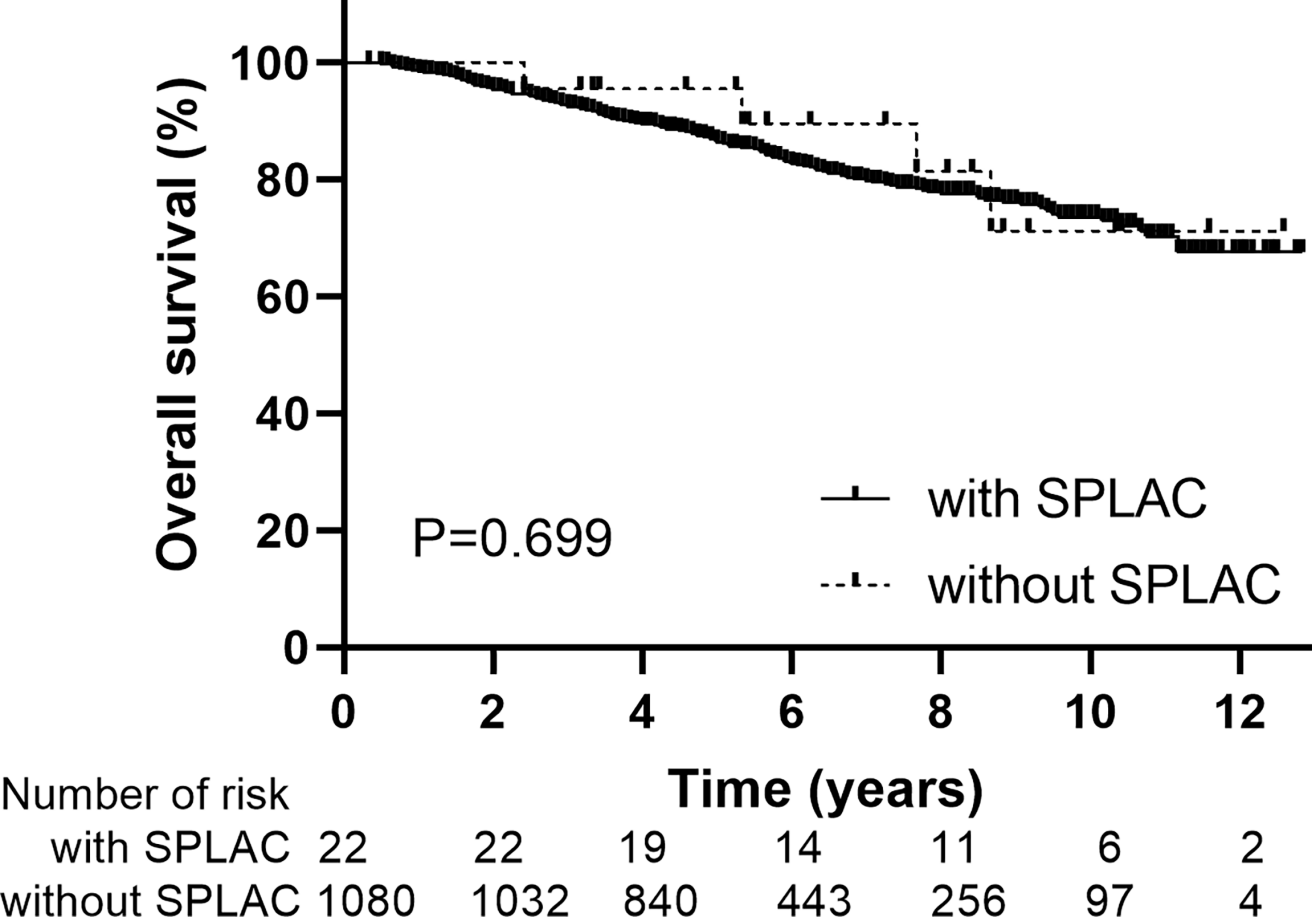
Overall survival for patients with or without second primary lung adenocarcinoma.

Of the 1,102 patients in this study, there are 809 men and 293 women. The median age at diagnosis of NPC was 50 years old (range, 18–78 years). According to AJCC eighth staging edition, there are 44, 222, 413, and 423 patients with stage I, II, III, and IVA disease, respectively. Most patients (928/1,102, 84.2%) received IMRT with chemotherapy and 174/1,102 (15.8%) received IMRT alone. The median follow-up duration for the whole group was 66 months (range, 4–154 months). The 5- and 10-year LFFS, RFFS, DMFS, and OS rates were 93.3% and 84.4%, 95.3% and 86.3%, 89.6% and 81.3%, and 86.6% and 73.4%, respectively. During follow-up, 22 SPLAC cases were observed with a crude incidence of 2.0% (22/1102). The baseline data were similar in age, sex, stage, and with or without chemotherapy for NPC patients with or without SPLAC (**Table 1**). The 10-year OS rates for NPC patients with or without SPLAC were 71.2% and 73.6% (P = 0.699), respectively (**Figure 1**).

**Table 1:** Baseline characteristics of NPC patients with or without SPLAC.

Characteristic	No. of patients	Without SPLAC	With SPLAC	P
Total patients	1,102	1,080	22	/
Age				0.103
≤50 years old	590	582	8	
>50 years old	512	498	14	
Gender				0.941
Male	809	793	16	
Female	293	287	6	
T category				0.227
T1	184	177	7	
T2	355	348	7	
T3	345	341	4	
T4	218	214	4	
N stage				0.062
N0	155	147	8	
N1	381	375	6	
N2	332	327	5	
N3	234	231	3	
Clinical stage				0.051
I	44	41	3	
II	222	215	7	
III	413	408	5	
IVA	423	416	7	
chemotherapy				0.74
No	174	167	7	
Yes	928	913	15	

NPC, nasopharyngeal carcinoma; SPLAC, second primary lung adenocarcinoma.

### Second Primary Lung Adenocarcinoma Incidence and Related Details

**Figure 2:**
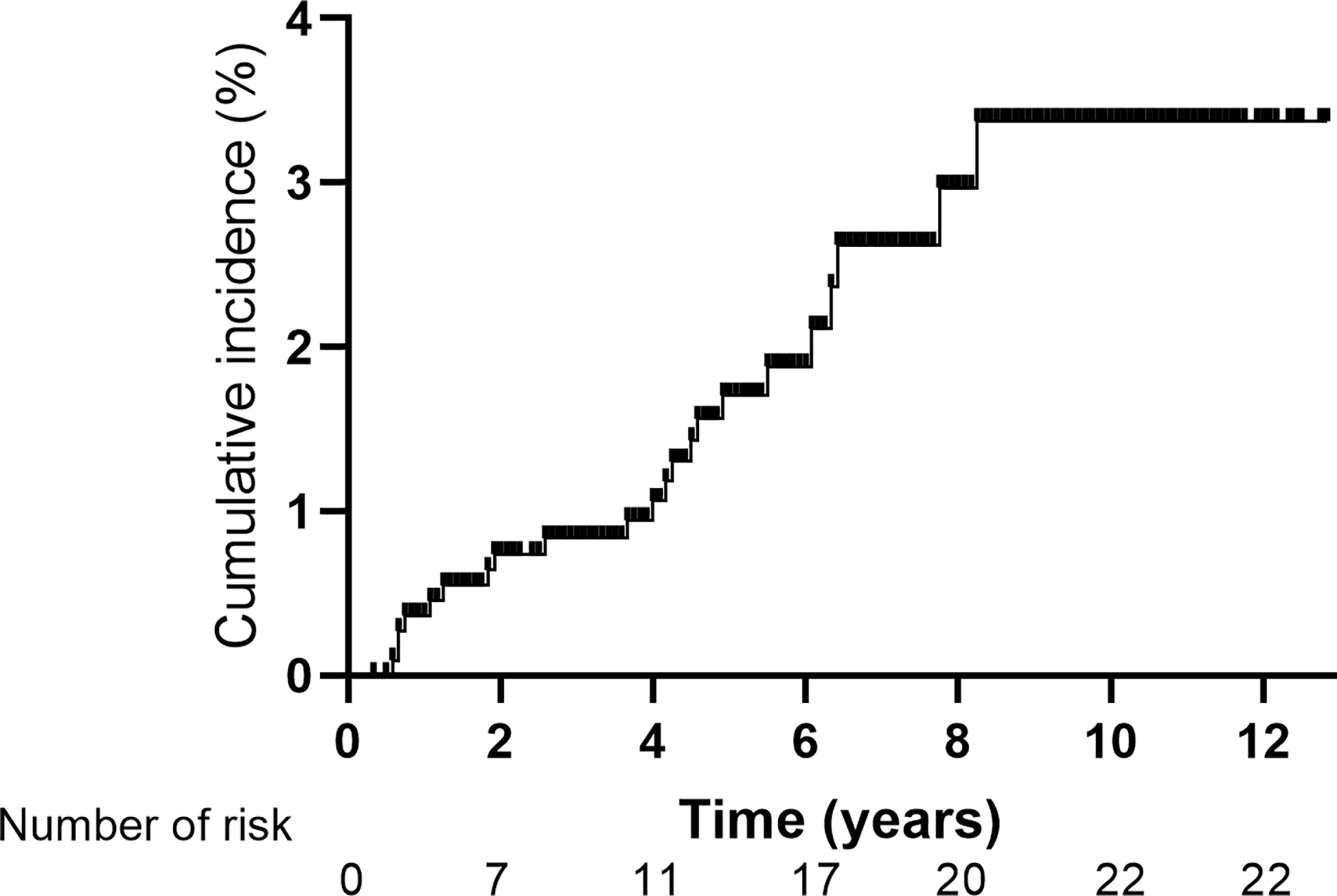
Cumulative incidence of second primary lung adenocarcinoma for 1,102 patients with non-metastatic nasopharyngeal carcinoma.

The median latency from the diagnosis of NPC to the diagnosis of SPLAC was 48 months (range, 7–99 months). The 1-, 2-, 3-, 4-, and 5-year cumulative risks of SPLAC were 0.4%, 0.7%, 0.8%, 1.1%, and 1.7%, respectively (**Figure 2**). Of the 22 patients, 16 (72.7%) developed SPLAC within 5 years and 6 (27.3%) developed SPLAC after 5 years. Male incidence and female incidence were similar, with 2.0% (16/809) and 2.0% (6/293). The age range of patients at diagnosis of SPLAC was 29–72 years old. Among them, 50.0% (3/6) female SPLAC patients and 12.5% (2/16) male SPLAC patients were ≤50 years old. During the follow-up, 200 patients died, of whom 4 (2.0%) died of SPLAC. The details of 22 SPLAC cases were shown in **Table 2**. Routine chest CT during follow-up detected pulmonary lesions of 5–9 mm in diameter in 10 patients, 10–14 mm in 6 patients, and 15–20 mm in 4 patients. Adenocarcinoma cells were found in 1 patient’s pleural effusion. Another patient was found with a burr lump of 42 * 37 mm in diameter at the apex of the left lung, lymph node metastasis to the left supraclavicular, mediastinum, and hilar, as well as brain metastasis (with puncture of pulmonary lump defined as adenocarcinoma). These two patients were unqualified for surgery. The lesions of 3 cases were in the apex of the lung, and 19 cases were in different pulmonary lobes. Among the 22 patients, 20 cases (90.9%) were stage 0/I and 19 patients underwent surgery with postoperative pathology-proven adenocarcinoma. Four cases received lobectomy, seven cases received segmentectomy, and eight cases received wedge resection. One patient refused surgery and received medication after biopsy of pulmonary nodule was confirmed as adenocarcinoma. One of the 19 patients died of SPLAC recurrence 84 months after operation, and the recurrence rate of surgery was 5.3% (1/19).

**Table 2:** Status of pulmonary lesions in SPLAC.

Patient No.	Location	Type	Size mm	Diagnosis months	Therapy	Response	Status	Survival months
**1**	Right middle lobe	GGO	<10	73	Surgery	Complete response	Alive	151
**2**	Right lobe	GGO	<10	13	Surgery	Complete response	Alive	139
**3**	Left superior lobe	solid nodule	<10	55	Surgery	Complete response	Alive	125
**4**	Right superior lobe	solid nodule	<10	99	Surgery	Complete response	Alive	106
**5**	Right superior lobe	GGO	<10	8	Surgery	Recurrence	Died	92
**6**	Right superior lobe	GGO	<10	66	Surgery	Complete response	Alive	97
**7**	Apex of left lung	GGO	<10	77	Surgery	Complete response	Alive	87
**8**	Left superior lobe	GGO	<10	54	Surgery	Complete response	Alive	75
**9**	Right superior lobe	GGO	<10	22	Surgery	Complete response	Alive	40
**10**	Right superior lobe	GGO	<10	15	Surgery	Complete response	Alive	41
**11**	Left superior lobe	solid nodule	10	31	Surgery	Complete response	Alive	128
**12**	Right superior lobe	GGO	10	59	Surgery	Complete response	Alive	101
**13**	Right superior lobe	GGO	10	48	Surgery	Complete response	Alive	65
**14**	Right middle lobe	GGO	10	23	Surgery	Complete response	Alive	68
**15**	Right superior lobe	GGO	12	76	Surgery	Complete response	Alive	110
**16**	Apex of left lung	GGO	14	7	Surgery	Complete response	Alive	38
**17**	Right superior lobe	GGO	15	51	Surgery	Complete response	Alive	124
**18**	Right inferior lobe	GGO	15	44	Surgery	Complete response	Alive	63
**19**	Right inferior lobe	GGO	15	8	Surgery	Complete response	Alive	55
**20**	Left superior lobe	solid nodule	20	50	Medication	Progression	Died	64
**21**	pleural effusion	/	/	9	Medication	Progression	Died	29
**22**	Apex, mediastinal lymph nodes	lump	42	93	Medication	Progression	Died	104

SPLAC, second primary lung adenocarcinoma; GGO, ground-glass opacity.

## Discussion

The second primary cancer was found in many patients with posttreatment primary malignancies during their follow-up, and the proportion of which is higher than that in the general population (9–13). Results of a retrospective study indicated that the overall risk of developing a second primary cancer is statistically significantly higher for 18 of 30 primary malignancies in men and 21 of 31 primary malignancies in women when compared with the general population (14). Song et al. (9) reported that among 2,285 patients with second primary lung cancer, the most common first primary malignancies were prostate cancer, breast cancer, bladder cancer, colorectal cancer, esophageal cancer, thyroid cancer, and cervical cancer. In general, the longer a life span is for primary malignancies after treatment, the more likely it is to develop a second primary cancer. The second primary cancer for NPC patients after radiotherapy was also reported by some previous studies (4, 6, 7, 15). Survivors of NPC patients had a higher risk of cancer than the general population. With a median follow-up of 10.8 years, a total of 290 cases of second primary cancer were observed in NPC patients treated at six centers in Hong Kong, with an incidence of 9.2% (290/3,166) (7). The most common second primary cancer was lung cancer (1.6%, 51/3,166), oral cancer, colorectal cancer, and bone and soft tissue tumors. During the follow-up, 1,188 patients died, of whom 114 (9.6%) died of the second primary malignancy. Zhang et al. (6) reported an incidence of 3.0% of second primary cancer for NPC patients, with 1-, 2-, 3-, 4-, and 5-year cumulative risks of 0.4%, 0.9%, 1.6%, 2.2%, and 2.6%, respectively. The median time from IMRT to the diagnosis of the second primary cancer was 37 months (6–102 months), with 14.3% patients within 1 year, 38.1% within 1–3 years, 33.9% within 3–5 years, and 13.7% after 5 years, respectively. Among them, lung cancer also had the highest incidence (50/6,377, 0.78%). The 5-year OS rates were 70.0% and 95.0% for NPC patients with or without the second primary cancer (P < 0.001), respectively. Similar to previous studies, the median latency from the diagnosis of NPC to the diagnosis of SPLAC was 48 months (range, 7–99 months) in our study, with 72.7% (16/22) patients within 5 years and 27.3% (6/22) patients after 5 years. The 1-, 2-, 3-, 4-, and 5-year cumulative risks of SPLAC were 0.4%, 0.7%, 0.8%, 1.1%, and 1.7%, respectively. Of 200 patients who died in this whole group, 2.0% (4/200) died of SPLAC. There was no difference in OS between NPC patients with or without SPLAC because of the good postoperative effect of early lung cancer (10-year OS: 71.2% vs. 73.6%, P = 0.699) in our study.

With the prevalence of chest LDCT screening, the likelihood of early detection of lung cancer increased. Two important studies were conducted in China on the results of LDCT screening. The first study was LDCT for high-risk individuals (16). From August 2013 to August 2014, 11,332 people (7,144 males and 4,188 females) were selected from Minhang Community of Shanghai. Screening results suggested 27 cases of primary lung cancer (0.24%), including 24 cases (0.21%) of adenocarcinoma, and 22 cases (81.48%) of stage 0/I lung cancer. The detection rate of primary lung cancer was 238.26 cases per 100,000 people/year. The second study was LDCT for regular health examination (17). From 2012 to 2018, lung cancer (pathologically confirmed) was detected in 179 (2.1%) of 8,392 hospital staff in six hospitals in China. The incidence rate was significantly higher in women than in men (2.5% vs. 1.3%, P = 0.001). The detection rates of lung cancer in age ≤40-year-old group, 40–55-year-old group, and >55-year-old group were 1.0%, 2.6%, and 2.9%, respectively. In the previous two screening studies in China, there was a high proportion of adenocarcinoma (92.6%–98.8%), a low proportion of squamous cell carcinoma (0.6%–7.4%), and a high proportion of early (stage 0/I) disease (81.5%–97.2%). These data were similar to those released by the Shanghai Municipal Center for Disease Control and Prevention and those released in the United States (18, 19). In this study, the incidence of SPLAC was 2.0% (22/1102) with a median follow-up interval of 66 months. There was no significant increase compared with the general population. The incidence rate in female was not lower than that in male (both 2.0%). Among 22 cases of SPLAC found in this group, 90.9% were in early stage, and the proportion of young female was higher than that of male (50.0% vs. 12.5%).

Commonly, 95.5% of lung cancer detected by CT was represented as ground-glass opacity (GGO). GGO may be benign lesions such as inflammation or bleeding, atypical adenomatoid hyperplasia, or lung cancer (17). In surveillance of NPC, follow-up of 4–6 months was suggested for newly discovered ≤5-mm GGO (20). Generally, benign GGO will decrease or disappear, while malignant GGO will persist or develop. Aggressive surgical treatment is necessary for lesions that are highly suspected to be invasive lung adenocarcinoma radiographically and for GGO with increased diameter or solid components during follow-up. As reported by our hospital (8), the proportion of benign lesions in all surgically removed lung GGO is less than 10%, and the surgical efficacy of the early SPLAC is similar to that of the first primary lung adenocarcinoma. Good postoperative prognosis for the SPLAC was found in those with controlled first primary tumor. Ko et al. (21) also reported similar 5-year OS for first primary lung adenocarcinoma and SPLAC (81.8% vs. 72.9%, P = 0.069). Different surgical approaches may affect the outcomes of early-stage lung adenocarcinoma. According to Shi et al. (22), the OS of patients with early-stage non-small cell lung cancer who underwent lobectomy/segmentectomy was higher than those who underwent wedge resection. However, regarding disease-free survival and relapse-free survival, the three surgical approaches showed no significant difference. Among 19 patients who received surgery in this study, only 1 patient died of lung cancer recurrence 84 months after wedge resection. The rest were alive with complete response.

The causative agent of the second primary tumors is unclear at present and may be related to family history, genetic defects, infection, chemotherapy, radiotherapy, hormones, alcohol, tobacco, environment, and so on (23). Epstein–Barr virus was identified to be the dominant contributor to NPC but was only identified in a very small proportion of the second primary tumors. Literature suggested that low-dose radiation may be associated with the second primary tumor (7). Compared with 2DRT and 3-dimensional conformal radiotherapy (3DCRT), it was reported that IMRT could improve local control and survival rates for NPC patients as well as reduce dose exposure to parotid glands, temporal lobes, and other organs at risk. As a result, the incidence of late toxicities such as dry mouth, trismus, and temporal lobe injury was significantly decreased in the IMRT group (24). Therefore, volumes of peripheral normal tissues (like oral cavity, neck, and so on) receiving low-dose radiation increased in the IMRT group and may contribute to the incidence of second primary tumors (25). Chow et al. (7) reported that 51 of the 290 second primary tumors were found in the head and neck in NPC patients treated with IMRT, with the highest incidence. Only 5%–15% of primary sarcomas occurred in the head and neck region (26). However, 21 (84%) of the 25 second primary sarcomas occurred in the head and neck region after IMRT in NPC patients. And 6 of the 51 second primary lung cancers occurred in the apex of the lung (7). From 1996 to 2002, Goggins et al. (27) analyzed the standardized incidence of the second primary tumor in all parts of NPC patients after 2DRT, which was consistent with that after IMRT (1.93 vs. 1.90). Also, the second oral cancer and lung cancer contributed to the highest incidence in both 2DRT and IMRT groups. The potentially negative effect of the wider low-dose zone in IMRT may be counteracted by the potentially negative effect of the larger high-dose zone of 2DRT or 3DCRT (28). Ardenfors et al. (29) made IMRT and CRT (conformal radiotherapy) plans for 10 head and neck patients, and the treatment plan data were obtained to calculate the risk of radiation-induced malignancy in four different tissues using different risk models. The results showed that the total lifetime risks of developing radiation-induced secondary tumor from CRT and IMRT were comparable and in the interval 0.9%–2.5%. The incidence of SPLAC after radiotherapy for NPC patients in this group was 2.0% (22/1102), which was similar to that reported by other authors and that reported in the general population (7, 16, 17). The proportion of SPLAC occurring in lung apex (3/22) in this group was also similar to that reported by literature (6/51) (7).

Limitations for this study include a single-center retrospective experience with a limited number of cases without a control cohort. Prospective multicenter studies are needed to confirm the result. Besides, literature showed that some biomarkers are important for lung cancer screening or detecting recurrence, such as circulating microRNAs (miRNAs), circulating tumor DNA (ctDNA), or methylation markers (30, 31). It may be also meaningful to investigate these biomarkers in SPLAC in the future when we have enough cases.

## Conclusion

In conclusion, the proportion of SPLAC after IMRT for NPC patients in our single-institution study was similar to that of the normal population. Most SPLACs were found in early stage with good surgical efficacy. Attributing to early detection of chest CT during routine follow-up, long-term survival of NPC patients with SPLAC is not inferior to those without SPLAC. Therefore, close surveillance of NPC survivors for SPLAC is warranted.

## Data Availability Statement

The raw data supporting the conclusions of this article will be made available by the authors without undue reservation.

## Ethics Statement

The studies involving human participants were reviewed and approved by the institutional review board of Fudan University Shanghai Cancer Center. The patients/participants provided their written informed consent to participate in this study.

## Author Contributions

FX and XN collected the data and finished the quality control of data. XH and CH provided the study concepts. FX, XN, and XH designed the study and performed the statistical analysis. FX and XH wrote the article. All authors contributed to the article and approved the submitted version.

## Funding

This work was supported by the Shanghai Sailing Program (grant no. 21YF1408400), Scientific and Innovative Action Plan of Shanghai (grant no. 21Y11911900), and institutional grant of Fudan University Shanghai Cancer Center (grant no. YJQN202023).

## Conflict of Interest

The authors declare that the research was conducted in the absence of any commercial or financial relationships that could be construed as a potential conflict of interest.

## Publisher’s Note

All claims expressed in this article are solely those of the authors and do not necessarily represent those of their affiliated organizations, or those of the publisher, the editors and the reviewers. Any product that may be evaluated in this article, or claim that may be made by its manufacturer, is not guaranteed or endorsed by the publisher.
